# Incidence and associated factors of venous thromboembolism in patients with gastric cancer across treatment settings after diagnosis: a systematic review and meta-analysis

**DOI:** 10.1186/s12959-026-00864-7

**Published:** 2026-04-21

**Authors:** Xing Jin, Zhiting Dong, Ningjing Tang, Qingyu Zhang, Jing Li

**Affiliations:** 1 Department of Anesthesiology, Affiliated Hospital of Beihua University, Jilin, Jilin Province China; 2 Department of Obstetrics and Gynecology, Jilin Maternity and Child Health Care Hospital (Jilin Obstetrics and Gynecology Hospital), Jilin, Jilin Province China; 3 Yanbian University School of Medicine, Yanji, Jilin Province China; 4 Department of Pain Medicine, Yanbian University Hospital, Yanji, Jilin Province China; 5 Department of Neurobiology, School of Basic Medical Sciences, Wannan Medical College, Wuhu, Anhui Province China

**Keywords:** Gastric cancer, Venous thromboembolism, Cancer-associated thrombosis, Incidence, Associated factors, Meta-analysis

## Abstract

**Background:**

Venous thromboembolism (VTE) is a major complication in patients with cancer. Although gastric cancer is recognized as a high-risk malignancy for VTE, existing evidence has mainly focused on perioperative settings. The burden of thrombotic events across broader treatment settings after diagnosis remains less well characterized. This study aimed to estimate the incidence of thrombotic events in patients with gastric cancer across treatment settings after diagnosis and to summarize reported associated factors.

**Methods:**

A systematic review and meta-analysis was conducted in accordance with PRISMA 2020 guidelines. PubMed, Embase, Web of Science, and the Cochrane Library were searched from inception to January 31, 2026. Studies reporting thrombotic event incidence and/or associated factors in patients with pathologically confirmed gastric cancer were included. Random-effects models were applied. Heterogeneity was assessed using the I² statistic. Sensitivity and exploratory subgroup analyses were performed where applicable.

**Results:**

Ten studies comprising 9,689 patients were included. In the primary analysis, the pooled incidence of thrombotic events across treatment settings after diagnosis was 9.0% (95% CI 6.1%–13.2%), with substantial heterogeneity (I² = 97.4%). Quantitative analyses of associated factors, including sex, body mass index ≥ 25 kg/m², peritoneal metastasis, lung metastasis, and bone metastasis, did not establish consistent and reproducible independent associations under random-effects models.

**Conclusions:**

Thrombotic risk appears clinically relevant in patients with gastric cancer across treatment settings after diagnosis, beyond short-term perioperative settings. However, current evidence does not establish robust and reproducible predictors for direct clinical implementation. Further large-scale prospective studies are needed to improve risk estimation and support future validation of risk assessment approaches in this population.

**Supplementary Information:**

The online version contains supplementary material available at 10.1186/s12959-026-00864-7.

## Introduction

Thrombotic events, particularly venous thromboembolism (VTE) comprising deep vein thrombosis (DVT) and pulmonary embolism (PE), are among the most common and clinically significant complications in patients with malignancies [1–3]. In individuals receiving anticancer therapy, the incidence of VTE is substantially higher than in the general population, ranging from approximately 4% to 20%, with risk influenced by tumor type, disease stage, and treatment exposure [1, 3]. Cancer-associated thrombosis (CAT) not only increases morbidity and mortality but has also been reported as the second leading cause of death, after cancer progression, among patients undergoing outpatient chemotherapy [4]. Moreover, VTE frequently leads to interruption or delay of anticancer treatment, thereby adversely affecting overall prognosis [1, 5].

Gastric cancer represents one of the most burdensome gastrointestinal malignancies worldwide. According to GLOBOCAN data, approximately 1.03 million new cases and 0.78 million deaths were reported globally in 2018 [6], and gastric cancer remained among the top three causes of cancer-related mortality in 2020 [7]. Previous studies have consistently identified gastric cancer as a high-risk tumor type for VTE, particularly among patients with advanced disease and those receiving systemic therapy [1, 3].

However, existing research on thrombotic complications in patients with gastric cancer has largely focused on perioperative populations, and available systematic reviews primarily reflect surgery-related short-term risk [8, 9]. These studies may not fully capture the burden of VTE across broader treatment settings after diagnosis. Systemic therapy has long been a central component of gastric cancer management, and many patients with advanced disease undergo multi-line and prolonged anticancer treatment [10]. The American Society of Clinical Oncology (ASCO) guidelines emphasize that VTE risk in cancer patients is dynamic and should be continuously assessed throughout the treatment course, with decisions regarding prophylactic anticoagulation individualized according to patient risk [5]. Nevertheless, anticoagulation therapy carries an inherent risk of bleeding, particularly in patients with gastrointestinal malignancies, warranting careful risk–benefit evaluation. Accordingly, a clearer understanding of VTE burden and the factors reported to be associated with its occurrence in gastric cancer patients across treatment settings after diagnosis remains clinically important.

Although several potential associated factors, including sex, body mass index (BMI), metastatic status, and laboratory markers, have been reported in previous studies [11], findings remain inconsistent. Most available evidence derives from single-center retrospective studies, and comprehensive quantitative synthesis is lacking. Moreover, it remains uncertain which factors show consistent and reproducible independent associations with VTE in this population.

Accordingly, we conducted a systematic review and meta-analysis to estimate the overall incidence of thrombotic events in patients with gastric cancer across treatment settings after diagnosis and to summarize the currently reported associated factors. When data permitted, we also performed quantitative synthesis of selected factors to explore their potential relevance for future risk assessment research, rather than for direct clinical implementation.

## Methods

This study was conducted as a systematic review and meta-analysis to comprehensively evaluate the incidence of thrombotic events in patients with gastric cancer across treatment settings after diagnosis, with particular emphasis on venous thromboembolism (VTE), and beyond studies primarily focused on surgery-related perioperative risk. When data permitted, quantitative synthesis of associated factors was also performed. The study design and reporting adhered to the Preferred Reporting Items for Systematic Reviews and Meta-Analyses (PRISMA) 2020 guidelines [12]. The study protocol was registered in the PROSPERO database (CRD420261303039). As this study was based on secondary analysis of published data and did not involve individual patient information, ethical approval was not required.

### Literature search

A systematic literature search was conducted in PubMed, Embase, Web of Science, and the Cochrane Library from database inception to January 31, 2026. The search was limited to studies published in English. The search strategy was constructed around two core concepts: gastric cancer (e.g., “gastric cancer,” “stomach cancer,” “gastric neoplasm,” “gastric carcinoma,” and related controlled vocabulary terms) and VTE (e.g., “venous thromboembolism,” “VTE,” “deep vein thrombosis,” “DVT,” “pulmonary embolism,” “PE,” and related controlled vocabulary terms), combined using Boolean operators. To maximize search sensitivity, no additional restriction block for incidence or associated factors was imposed during database retrieval. The full database-specific search strategies are provided in Supplementary Tables 2 to ensure reproducibility.

In addition, the reference lists of all included studies were manually screened to identify potentially relevant articles that may have been missed in the database search.

Given that the primary objectives of this study included extraction of quantifiable effect estimates (e.g., hazard ratios [HRs] and odds ratios [ORs]) for quantitative synthesis, gray literature (such as conference abstracts, unpublished studies, or dissertations) was not systematically searched. Such sources often lack sufficient methodological detail and extractable data for quantitative synthesis and formal risk-of-bias assessment.

### Eligibility criteria

Eligible studies included patients with pathologically confirmed gastric cancer and reported the occurrence of thrombotic events during follow-up after diagnosis or during anticancer treatment. The primary thrombotic outcome of interest was VTE, defined as deep vein thrombosis (DVT) and/or pulmonary embolism (PE), diagnosed according to imaging modalities or standardized clinical criteria as specified in each study. Studies that additionally included visceral vein thrombosis (IVT) within composite thrombotic outcomes were also considered eligible and were examined separately in subgroup analyses. Studies that reported symptomatic and/or incidental thrombotic events were both considered eligible, depending on the original study definition.

Studies were eligible if they reported thrombotic event incidence and/or evaluated at least one factor associated with thrombotic event occurrence. These factors included demographic characteristics (e.g., sex, age, body mass index [BMI]), tumor-related factors (e.g., stage, metastatic status), treatment-related variables, and established risk scores or laboratory markers. Eligible study designs comprised prospective or retrospective cohort studies, registry-based studies, and post hoc analyses of randomized controlled trials (RCTs). Case reports, case series, reviews, commentaries, animal studies, and studies without extractable outcome data or effect estimates were excluded.

Because the perioperative period is variably defined across the literature and lacks a universally accepted temporal boundary, we did not apply a single fixed postoperative time cutoff to define perioperative studies. Instead, we excluded studies primarily focused on surgery-centered perioperative VTE risk, particularly those evaluating predominantly operative or immediate postoperative predictors, while retaining studies that assessed thrombotic event occurrence in broader treatment settings after diagnosis.

### Study selection and data extraction

Study selection and data extraction were independently performed by two investigators. Discrepancies were resolved through discussion, with arbitration by a third investigator when necessary.

Extracted data included first author, publication year, country or region, study design, sample size, number of thrombotic events and cumulative incidence, population characteristics, VTE definition and diagnostic methods, follow-up period, and reported effect estimates for associated factor analyses (with preference given to multivariable-adjusted HRs, ORs, or relative risks [RRs]) along with their corresponding 95% confidence intervals (CIs). Data on follow-up definition, time origin, observation window, and outcome definition, including whether symptomatic and/or incidental VTE events were reported, were also extracted when available. When multiple adjusted models were reported, the estimate from the most fully adjusted model was preferentially extracted. Because cumulative incidence estimates are sensitive to differences in follow-up duration, time origin, and observation framework, these study-level definitions were taken into account when interpreting pooled incidence results.

### Quality assessment and risk of bias

The methodological quality of observational studies was assessed using the Newcastle–Ottawa Scale (NOS) [13], which evaluates selection of study groups, comparability, and outcome assessment. Randomized controlled trials were evaluated using the Cochrane Risk of Bias 2.0 tool [14]. All quality assessments were conducted independently by two investigators.

### Statistical analysis

All statistical analyses were performed using R software (version 4.3.2; R Foundation for Statistical Computing, Vienna, Austria), primarily employing the “meta” and “metafor” packages.

For the pooled analysis of thrombotic event incidence, an inverse-variance–weighted random-effects model was applied. Between-study variance (τ²) was estimated using the restricted maximum likelihood (REML) method. Pooled incidence estimates were presented as proportions with corresponding 95% confidence intervals (CIs). Statistical heterogeneity was assessed using Cochran’s Q test and the I² statistic. To reflect the potential range of true effects that may be observed in future comparable studies, 95% prediction intervals (PIs) were additionally calculated. Because pooled cumulative incidence estimates may be influenced by differences in follow-up duration, time origin, and observation framework, these study-level characteristics were taken into account when interpreting the pooled results.

In the analysis of associated factors, multivariable-adjusted effect estimates were preferentially extracted. Because different studies reported different types of effect measures, hazard ratios (HRs) and odds ratios (ORs) were pooled separately. Quantitative synthesis was performed only when at least three studies reported the same type of effect estimate for a given factor. Effect sizes were combined using inverse-variance–weighted random-effects models, with between-study variance (τ²) estimated via the REML method. Pooled effect estimates were reported with corresponding 95% CIs. HRs and ORs were synthesized separately rather than combined in a single meta-analysis.

To evaluate the robustness of the findings, leave-one-out sensitivity analyses were conducted for the primary analysis and key associated factors. Fixed-effect models were also applied for comparison. When data permitted, subgroup analyses by geographic region were performed to explore potential sources of heterogeneity. Given the limited number of studies available for several analyses, subgroup exploration was considered exploratory.

When at least ten studies were available for a given analysis, publication bias and small-study effects were planned to be assessed using funnel plots and Egger’s regression test. If fewer than ten studies were available, formal assessment of publication bias was not performed because such methods are generally considered unreliable with a limited number of studies. All statistical tests were two-sided, and P values < 0.05 were considered statistically significant.

## Results

### Study selection

A total of 2,807 records were identified through database searching, including PubMed (*n* = 498), the Cochrane Library (*n* = 85), Embase (*n* = 1,719), and Web of Science (*n* = 505). After removal of 1,116 duplicate records, 1,691 articles underwent title and abstract screening. Of these, 1,624 records were excluded, mainly because the population or outcome was not relevant, no extractable data on thrombotic event incidence or associated factors were available, or the articles were non-original publications.

**Fig. 1:**
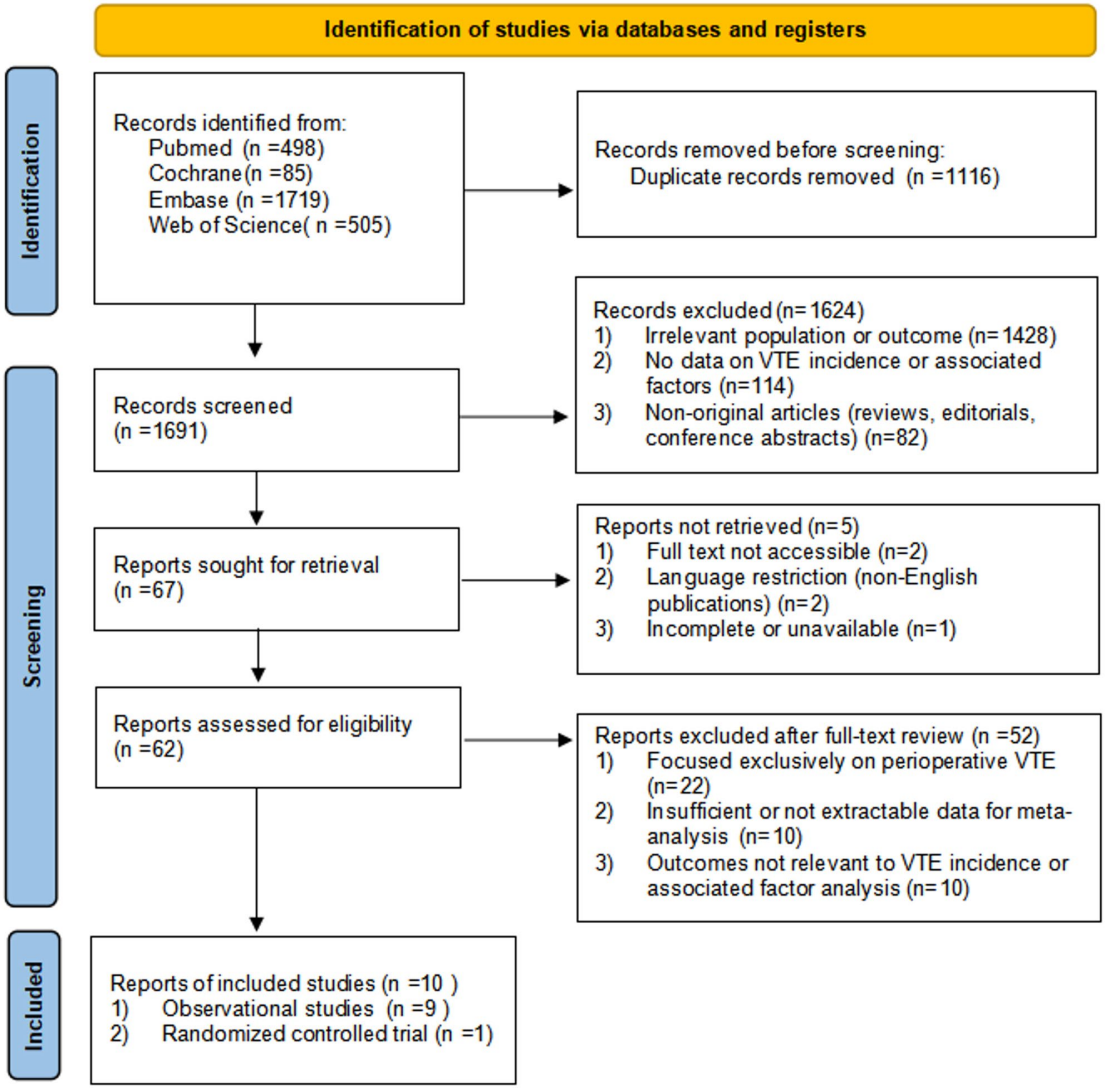
PRISMA flow diagram of study selection

Subsequently, 67 reports were sought for retrieval, of which 5 were not retrieved. A total of 62 full-text articles were assessed for eligibility. After full-text review, 52 studies were excluded, primarily because they focused exclusively on perioperative VTE, lacked sufficient extractable data for meta-analysis, or reported outcomes not relevant to thrombotic event incidence or associated factor analysis. Ultimately, 10 studies met the inclusion criteria and were included in the systematic review and quantitative synthesis (Fig. 1).

### Study characteristics and quality assessment

The included studies were published between 2009 and 2025 and comprised a total of 9,689 patients with gastric cancer. Most studies were retrospective cohort studies (*n* = 9), and one study was a post hoc analysis of a randomized controlled trial. Detailed characteristics of the included studies are presented in Table 1.

**Table 1:** Characteristics of included studies

First author (year)	Country/Region	Study design	VTE events / total (%)	Population characteristics	VTE definition	Diagnostic method	Follow-up period
Arai (2017)	Japan	Retrospective cohort study	37/281 (13.2%)	Advanced gastric cancer receiving first-line chemotherapy	DVT, PE	CT	During chemotherapy
Park (2017)	South Korea	Retrospective cohort study	27/241 (11.2%)	Advanced gastric cancer receiving palliative chemotherapy	DVT, PE	Doppler US, CT, CTA	Median follow-up: 10.8 months
Fuentes (2017)	USA	Retrospective cohort study	13/112 (11.6%)	All stages; mixed treatment modalities	DVT, PE, IVT	Doppler US, CT, CTA, V/Q scan	Median follow-up: 21.3 months
Lee (2009)	South Korea	Retrospective cohort study	73/2015 (3.6%)	All stages; majority underwent surgery	DVT, PE, IVT	Doppler US, CT, CTA, V/Q scan	Median follow-up: 23.2 months
Yoshikawa (2023)	Japan	Prospective cohort study	131/1896 (7.1%)	Stage II–IV receiving anti-tumor therapy	DVT, PE	Doppler US, CTA	Median follow-up: 11.4 months
Abdel-Razeq (2020)	Jordan	Retrospective cohort study	150/671 (22.4%)	Pathologically confirmed gastric adenocarcinoma	DVT, PE	Doppler US, CTA	Fixed follow-up: 12 months
Kang (2011)	South Korea	Retrospective cohort study	103/3095 (3.4%)	Unresectable advanced gastric cancer receiving chemotherapy	DVT, PE	Doppler US, CT, CTA, V/Q scan	Median time to VTE: 4.6 months
Slagter (2020)	Netherlands	Randomized controlled trial (secondary analysis)	78/781 (10.0%)	Resectable gastric cancer receiving neoadjuvant chemotherapy	DVT, PE, IVT	CT (symptom- or protocol-based)	During neoadjuvant therapy
Takayoshi (2019)	Japan	Retrospective cohort study	34/188 (18.1%)	Unresectable advanced gastric cancer	DVT, PE, IVT	CT, Doppler US	During chemotherapy
Turkoglu (2025)	Turkey	Single-center retrospective cohort study	21/337 (6.2%)	Metastatic gastric cancer receiving ≥ 1 line systemic therapy	DVT, PE	Imaging + clinical confirmation	From treatment initiation to death or last follow-up

Abbreviations: VTE, venous thromboembolism; DVT, deep vein thrombosis; PE, pulmonary embolism; IVT, intra-abdominal/visceral vein thrombosis; US, ultrasonography; CT, computed tomography; CTA, computed tomography angiography; V/Q scan, ventilation–perfusion scan; RCT, randomized controlled trial

Notes: Thrombotic outcomes included deep vein thrombosis (DVT) and/or pulmonary embolism (PE) in all studies; some studies additionally included intra-abdominal/visceral vein thrombosis (IVT) within composite thrombotic outcomes. Studies reporting splanchnic or visceral vein thrombosis (e.g., portal, splenic, or mesenteric vein thrombosis) were categorized as including IVT. Follow-up period refers to the risk window or observation period defined in each original study

The included studies covered heterogeneous clinical settings after diagnosis, including systemic therapy–related cohorts, mixed treatment settings, and broader follow-up populations. All studies captured DVT and/or PE, although some additionally included visceral vein thrombosis (IVT) within composite thrombotic outcomes. Diagnostic methods also varied across studies. Follow-up definitions differed substantially, ranging from treatment-related observation windows (e.g., during chemotherapy or neoadjuvant therapy) to predefined follow-up intervals such as 12 months, as well as longer observational periods reported by median follow-up duration. The included populations were also heterogeneous with respect to disease stage and treatment setting, including studies of advanced disease, mixed-stage cohorts, and patients receiving different anticancer treatment modalities. In addition, outcome definitions were not fully uniform across studies, and the inclusion of symptomatic and incidental events varied according to the original study design and reporting.

The methodological quality of observational studies was assessed using the Newcastle–Ottawa Scale (NOS), with most studies scoring between 7 and 9 points, indicating moderate to high quality (Table 2). The randomized controlled trial was evaluated using the Cochrane Risk of Bias 2.0 tool and was judged to have “some concerns” regarding overall risk of bias (Table 3).

**Table 2:** Risk of bias assessment of observational studies using the newcastle–ottawa scale (NOS)

No.	First Author (Year)	Selection (maximum 4 points)				Comparability (maximum 2 points)		Outcome (maximum 3 points)			Total Score (9 points)	Quality Category
		Representativeness of the exposed cohort	Selection of the non-exposed cohort	Ascertainment of exposure	Demonstration that outcome of interest was not present at start of study	Comparability of cohorts on the basis of the design or analysis	Additional control for important factor or additional factor	Assessment of outcome	Was follow-up long enough for outcomes to occur	Adequacy of follow-up of cohorts		
1	Arai (2017)	★	★	★	★	★		★	★		7	High
2	Park (2017)	★	★	★	★	★	★	★	★	★	7	High
3	Fuentes (2018)	★	★	★	★	★	★	★	★		8	High
4	Lee (2009)	★	★	★	★	★	★	★	★	★	9	High
5	Yoshikawa (2023)	★	★	★	★	★	★	★	★	★	9	High
6	Abdel-Razeq (2020)	★	★	★		★		★	★		6	Moderate
7	Kang (2011)	★	★	★		★	★	★	★	★	8	High
8	Takayoshi (2019)	★	★	★		★		★	★	★	7	High
9	Turkoglu (2025)	★	★	★	★	★		★	★	★	8	High

Note. A star (★) indicates that one point was awarded for the corresponding item. Blank cells indicate that the criterion was not met or not reported. Studies scoring 7–9 points were considered high quality, 5–6 moderate quality, and ≤ 4 low quality

**Table 3:** Risk of bias assessment of the randomized controlled trial by Slagter et al. (2020) using the ROB 2.0 tool

Domain	Risk of Bias Judgment	Support for Judgment
D1: Randomization process	Some concerns	Subanalysis of CRITICS trial; central randomization used but details limited in this report
D2: Deviations from intended interventions	Low	Open-label design; completion rates balanced between groups; objective VTE outcome
D3: Missing outcome data	Low	Complete follow-up (781/781 analyzed); no missing outcome data
D4: Measurement of outcome	Low	VTE defined by standardized imaging; objective endpoint not influenced by knowledge of intervention
D5: Selection of reported result	Some concerns	VTE was adverse event, not primary endpoint; risk factor analysis not pre-specified
Overall risk of bias	Some concerns	

### Meta-analysis of thrombotic event incidence in patients with gastric cancer (primary analysis)

Ten studies [11, 15–23], comprising a total of 9,689 patients with gastric cancer, were included in the primary incidence analysis. Across these studies, thrombotic outcomes were variably defined as VTE alone (DVT and/or PE) or as VTE plus visceral vein thrombosis (IVT). The reported incidence across individual studies ranged from 3.3% to 22.4%.

**Fig. 2:**
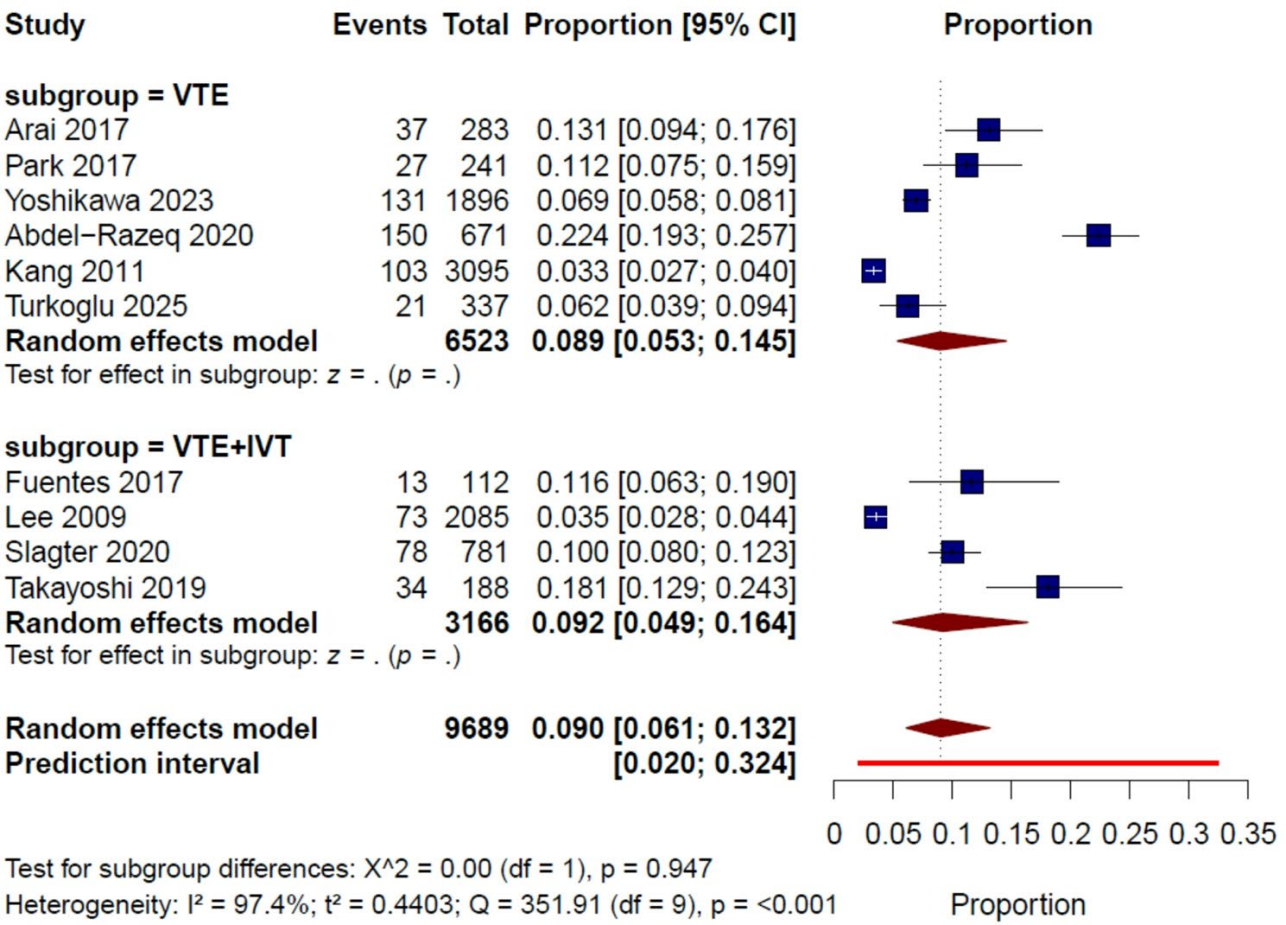
Pooled incidence of thrombotic events in patients with gastric cancer across treatment settings after diagnosis, with subgroup analysis by outcome definition

Using a random-effects model, the pooled incidence of thrombotic events across treatment settings after diagnosis was 9.0% (95% CI, 6.1%–13.2%). Substantial between-study heterogeneity was observed (I² = 97.4%, τ² = 0.4403, Q = 351.91, *P* < 0.001). The 95% prediction interval ranged from 2.0% to 32.4% (Fig. 2).

#### Subgroup analysis by outcome definition

In an exploratory subgroup analysis stratified by outcome definition, the pooled incidence was 8.9% (95% CI, 5.3%–14.5%) among the six studies defining outcomes as VTE alone, and 9.2% (95% CI, 4.9%–16.4%) among the four studies that additionally included IVT. No statistically significant difference was observed between subgroups (*P* = 0.947) (Fig. 2).

#### Subgroup analysis of VTE incidence by geographic region

**Fig. 3:**
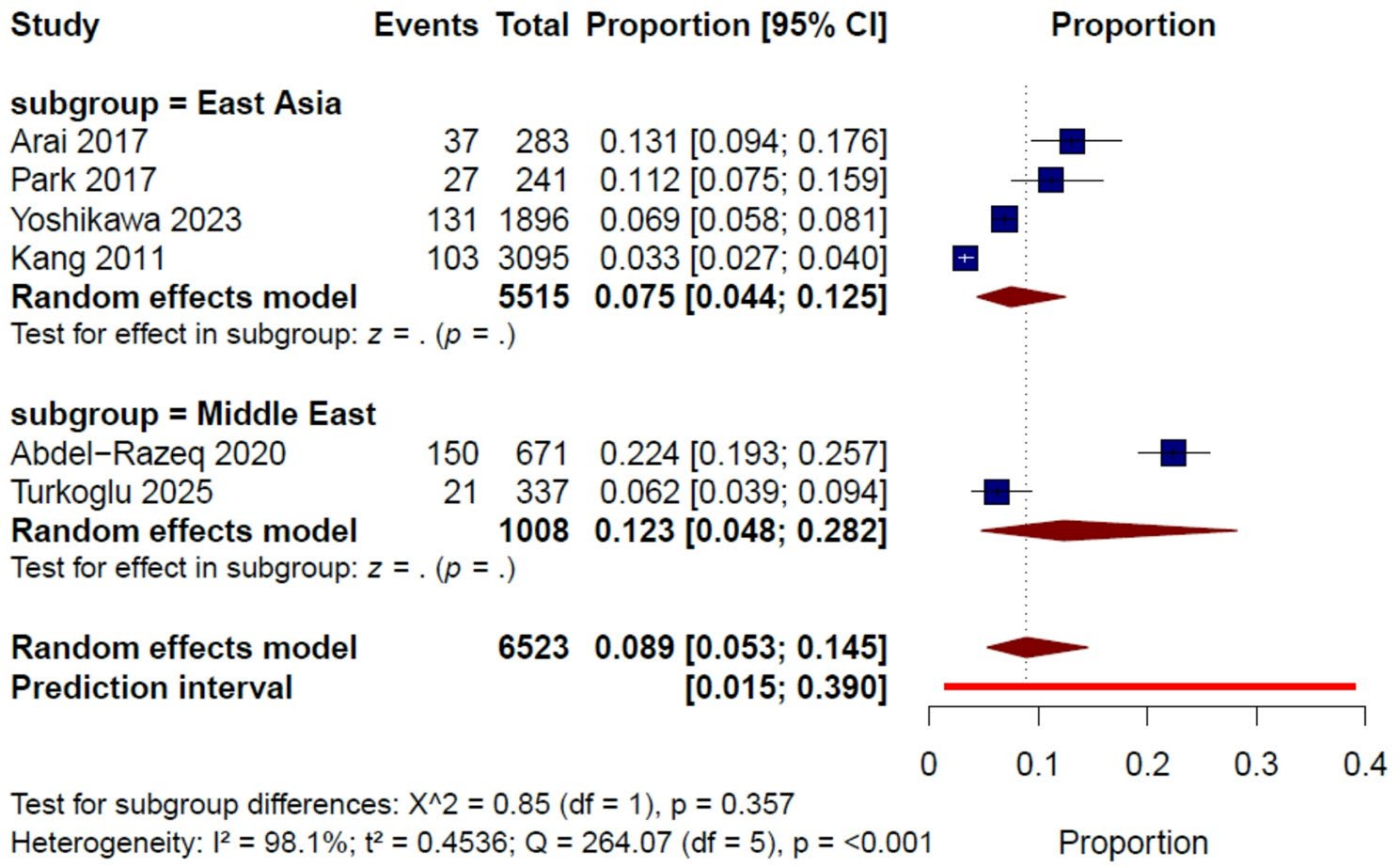
Pooled incidence of venous thromboembolism in patients with gastric cancer, with subgroup analysis by geographic region

Among the six studies defining thrombotic outcomes as VTE alone, the pooled incidence of VTE was 8.9% (95% CI, 5.3%–14.5%) with substantial heterogeneity (I² = 98.1%, τ² = 0.4536, Q = 264.07, *P* < 0.001). The 95% prediction interval for this pooled estimate ranged from 1.5% to 39.0%. In an exploratory subgroup analysis by geographic region, the pooled incidence was 7.5% (95% CI, 4.4%–12.5%) in East Asia and 12.3% (95% CI, 4.8%–28.2%) in the Middle East. The between-subgroup difference was not statistically significant (*P* = 0.357) (Fig. 3). Given the limited number of studies within each subgroup and the substantial residual heterogeneity, these subgroup findings should be interpreted cautiously.

#### Sensitivity analyses

In the leave-one-out sensitivity analysis, the pooled incidence ranged from 7.3% to 11.0%, with no substantial change in the overall estimate (Supplementary Figure S1). Using a fixed-effect model, the pooled incidence was 8.9% (95% CI, 8.2%–9.7%), which was consistent in direction with the random-effects model (Supplementary Figure S2).

### Quantitative meta-analysis of associated factors

#### Sex (female vs. male)

**Fig. 4:**
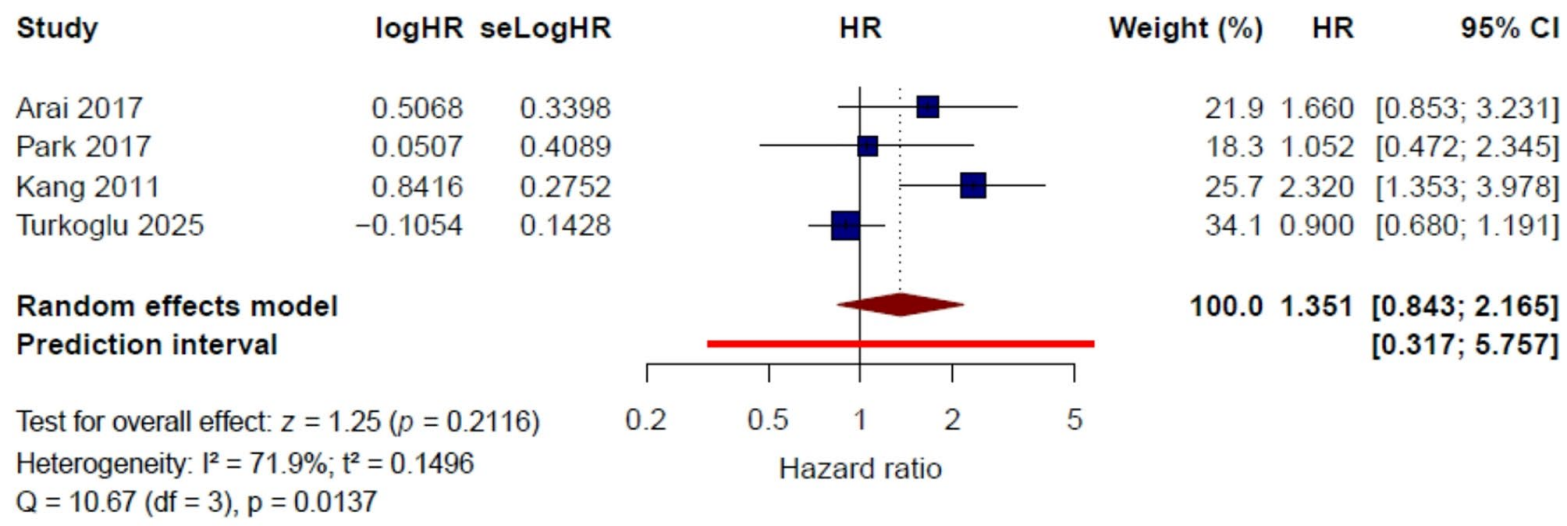
Female sex and risk of venous thromboembolism: pooled multivariable hazard ratios

Four studies [11, 15, 20, 23] were included in the quantitative synthesis for sex. Under the random-effects model, female sex was not significantly associated with thrombotic risk in patients with gastric cancer (HR = 1.35, 95% CI 0.84–2.17, *P* = 0.212). Substantial heterogeneity was observed (I² = 71.9%, τ² = 0.1496, Q = 10.67, *P* = 0.014) (Fig. 4).

In the leave-one-out sensitivity analysis, the pooled HR ranged from 1.06 to 1.72, indicating that the overall estimate was sensitive to study composition (Supplementary Figure S3). After exclusion of Kang et al. [20], heterogeneity decreased substantially (I² = 28.1%), and the pooled HR was 1.06 (95% CI 0.74–1.50). After exclusion of Turkoglu et al. [23], heterogeneity also decreased (I² = 23.7%), and the pooled HR became 1.72 (95% CI 1.12–2.65), suggesting that the overall result was influenced by this study.

Using a fixed-effect model, the pooled HR was 1.15 (95% CI 0.92–1.44, *P* = 0.225) (Supplementary Figure S4). Overall, these findings do not support a consistent and reproducible independent association between female sex and thrombotic risk in this population.

#### BMI ≥ 25 kg/m²

**Fig. 5:**
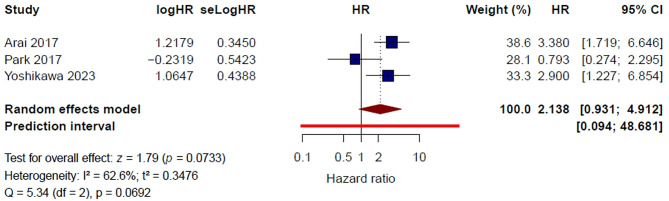
Body Mass Index ≥ 25 kg/m² and risk of venous thromboembolism: pooled multivariable hazard ratios

Three studies [11, 15, 23] were included in the quantitative synthesis for BMI ≥ 25 kg/m². Under the random-effects model, BMI ≥ 25 kg/m² was not significantly associated with thrombotic risk in patients with gastric cancer (HR = 2.14, 95% CI 0.93–4.91, *P* = 0.073). Moderate heterogeneity was observed (I² = 62.6%, τ² = 0.3476, Q = 5.34, *P* = 0.069) (Fig. 5). The 95% prediction interval ranged from 0.09 to 48.68, indicating substantial uncertainty in the expected effect across comparable study settings.

In the leave-one-out sensitivity analysis, the pooled HR ranged from 1.58 to 3.19, indicating that the overall estimate was sensitive to study composition (Supplementary Figure S5). After exclusion of Park et al. [15], the pooled HR increased to 3.19 (95% CI 1.87–5.42) and heterogeneity decreased to 0%, suggesting that this study substantially influenced the overall result. In contrast, omission of Arai et al. [11] or Yoshikawa et al. [23] did not materially alter the direction of the association, although the pooled estimate remained imprecise.

Using a fixed-effect model, the pooled HR was 2.41 (95% CI 1.50–3.88, *P* = 0.0003) (Supplementary Figure S6). Overall, these findings suggest a possible positive association between BMI ≥ 25 kg/m² and thrombotic risk, but the between-study heterogeneity and sensitivity to study exclusion indicate that the evidence is not yet sufficiently robust to support a consistent and reproducible independent association.

#### Peritoneal metastasis (Yes vs. No)

**Fig. 6:**
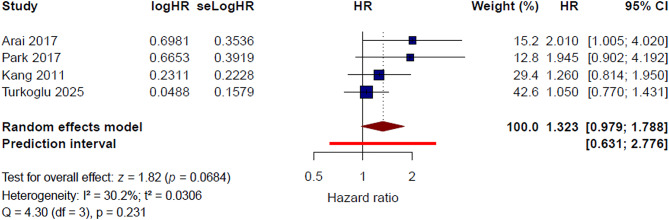
Peritoneal metastasis and risk of venous thromboembolism: pooled multivariable hazard ratios

Four studies [11, 15, 20, 23] were included in the quantitative synthesis for peritoneal metastasis. Under the random-effects model, peritoneal metastasis was not significantly associated with thrombotic risk in patients with gastric cancer (HR = 1.32, 95% CI 0.98–1.79, *P* = 0.068). Low-to-moderate heterogeneity was observed (I² = 30.2%, τ² = 0.0306, Q = 4.30, *P* = 0.231) (Fig. 6). The 95% prediction interval ranged from 0.63 to 2.78, indicating uncertainty in the expected effect across comparable study settings.

In the leave-one-out sensitivity analysis, the pooled HR ranged from 1.18 to 1.54 (Supplementary Figure S7). After exclusion of Turkoglu et al. [23], the pooled HR increased to 1.54 (95% CI 1.08–2.18) and heterogeneity decreased to 0%, suggesting that the overall estimate was influenced by this study. By contrast, exclusion of the other individual studies did not materially alter the direction of the association, although the pooled estimates remained statistically non-significant.

Using a fixed-effect model, the pooled HR was 1.25 (95% CI 0.99–1.57, *P* = 0.056) (Supplementary Figure S8). Overall, these findings suggest a possible positive association between peritoneal metastasis and thrombotic risk, but the evidence remains insufficient to support a consistent and reproducible independent association.

#### Lung metastasis (Yes vs. No)

**Fig. 7:**
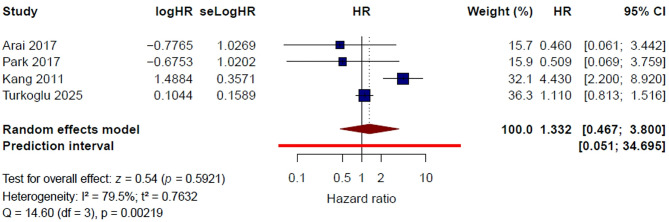
Lung metastasis and risk of venous thromboembolism: pooled multivariable hazard ratios

Four studies [11, 15, 20, 23] were included in the quantitative synthesis for lung metastasis. Under the random-effects model, lung metastasis was not significantly associated with thrombotic risk in patients with gastric cancer (HR = 1.33, 95% CI 0.47–3.80, *P* = 0.592). Substantial heterogeneity was observed (I² = 79.5%, τ² = 0.7632, Q = 14.60, *P* = 0.002) (Fig. 7). The 95% prediction interval ranged from 0.05 to 34.70, indicating marked uncertainty in the expected effect across comparable study settings.

In the leave-one-out sensitivity analysis, the pooled HR ranged from 1.07 to 1.62 (Supplementary Figure S9). After exclusion of Kang et al. [20], heterogeneity decreased to 0%, and the pooled HR was 1.07 (95% CI 0.79–1.45), suggesting that this study substantially influenced the observed between-study heterogeneity. Exclusion of the other individual studies did not materially alter the overall non-significant association.

Using a fixed-effect model, the pooled HR was 1.34 (95% CI 1.01–1.77, *P* = 0.040) (Supplementary Figure S10). Overall, these findings suggest that the association between lung metastasis and thrombotic risk is sensitive to the choice of analytic model and to study composition, and therefore does not support a consistent and reproducible independent association.

#### Bone metastasis (Yes vs. No)

**Fig. 8:**
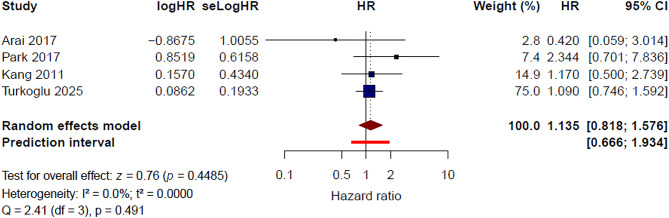
Bone metastasis and risk of venous thromboembolism: pooled multivariable hazard ratios

Four studies [11, 15, 20, 23] were included in the quantitative synthesis for bone metastasis. Under the random-effects model, bone metastasis was not significantly associated with thrombotic risk in patients with gastric cancer (HR = 1.14, 95% CI 0.82–1.58, *P* = 0.449). No significant heterogeneity was observed (I² = 0.0%, τ² = 0.0000, Q = 2.41, *P* = 0.491) (Fig. 8). The 95% prediction interval ranged from 0.67 to 1.93, suggesting limited between-study variability in the expected effect across comparable study settings.

In the leave-one-out sensitivity analysis, the pooled HR ranged from 1.07 to 1.28, with no meaningful change in the overall direction or significance of the association (Supplementary Figure S11). Heterogeneity remained low or absent across leave-one-out analyses, further indicating the stability of the pooled estimate.

Using a fixed-effect model, the pooled HR was identical to that of the random-effects model (HR = 1.14, 95% CI 0.82–1.58) (Supplementary Figure S12). Overall, these findings do not support a consistent independent association between bone metastasis and thrombotic risk in this population.

### Narrative synthesis of other risk factors

#### Age

Ten studies [11, 15–23] evaluated the association between age and VTE risk. Due to differences in age categorization and statistical modeling approaches, quantitative pooling was not performed. Most studies did not report a statistically significant association between age and VTE risk, although isolated studies suggested a possible increase in risk among older patients. Overall, findings were inconsistent.

#### Tumor stage

Three studies [16–18] assessed the relationship between tumor stage and VTE risk. Some studies reported a higher risk in advanced-stage disease, whereas others did not observe a statistically significant association. Given the heterogeneity in stage classification and reporting, quantitative pooling was not conducted.

#### History of surgery

Five studies [15–17, 20, 22] evaluated prior surgical history in relation to VTE risk. Results were inconsistent across studies, with some reporting an increased risk and others finding no statistically significant association.

#### Khorana risk score (KRS)

Four studies [16, 19, 22–23] examined the association between the Khorana Risk Score and VTE risk. Current evidence did not demonstrate a consistent or statistically significant association between higher KRS and VTE occurrence in patients with gastric cancer.

## Discussion

This systematic review and meta-analysis synthesized the available evidence on thrombotic events in patients with gastric cancer across treatment settings after diagnosis. In the primary incidence analysis including 10 studies, the pooled incidence of thrombotic events was 9.0%, although substantial between-study heterogeneity was observed. In the subgroup analysis by outcome definition, the pooled incidence was similar between studies reporting VTE alone and those additionally including visceral vein thrombosis (IVT), with no statistically significant subgroup difference. Among the six studies defining outcomes as VTE alone, the pooled incidence of VTE was 8.9%, and the exploratory subgroup analysis by geographic region did not identify a significant difference between East Asia and the Middle East. Taken together, these findings suggest that thrombotic risk in gastric cancer remains clinically relevant beyond studies limited to short-term perioperative settings, but the pooled incidence estimates should be interpreted cautiously because of heterogeneous follow-up frameworks, treatment settings, and outcome definitions.

Previous systematic reviews in gastric cancer have mainly focused on perioperative populations and surgery-related short-term thrombotic risk [8, 9]. By contrast, the present review aimed to address thrombotic burden across broader treatment settings after diagnosis, rather than focusing primarily on operative or immediate postoperative risk. This distinction is clinically important because the prothrombotic burden of gastric cancer is not limited to a single surgery-centered time window. Instead, it likely reflects a combination of tumor biology, metastatic burden, treatment exposure, and supportive care practices across different phases of management. At the same time, because the included studies differed substantially in follow-up duration, time origin, and observation windows, the pooled incidence estimates reported here should not be interpreted as fixed-time risks directly applicable to all clinical scenarios.

An important finding of this review is that no factor demonstrated a clearly consistent and reproducible independent association with thrombotic risk across studies. Female sex, BMI ≥ 25 kg/m², peritoneal metastasis, lung metastasis, and bone metastasis were quantitatively synthesized, but the pooled estimates were either non-significant under the random-effects model or sensitive to study composition, model choice, or both. For example, BMI ≥ 25 kg/m² and peritoneal metastasis showed possible positive associations in some sensitivity or fixed-effect analyses, whereas lung metastasis became significant only under the fixed-effect model despite substantial heterogeneity in the random-effects model. In contrast, bone metastasis showed relatively stable and non-significant pooled estimates across analytic approaches. Overall, these findings suggest that the currently available evidence is hypothesis-generating rather than sufficient to support direct clinical implementation on the basis of any single reported factor.

The substantial heterogeneity observed in the incidence analyses likely reflects multiple sources. First, VTE ascertainment strategies varied across studies, including symptom-triggered evaluation, partial screening approaches, and differences in imaging use, all of which may influence event detection. Second, the included populations were clinically heterogeneous with respect to disease stage, treatment setting, and exposure to multimodal management. Some studies focused predominantly on advanced disease or systemic therapy, whereas others included broader or mixed treatment settings. Third, outcome definitions were not fully uniform, particularly regarding whether thrombotic outcomes were restricted to DVT and PE or also included IVT, and whether symptomatic and incidental events were both captured. Fourth, variation in follow-up duration, time origin, and observation framework further limited the direct comparability of pooled cumulative incidence estimates. These issues should be considered when interpreting both the pooled incidence and the associated factor analyses.

From a biological perspective, the risk of cancer-associated thrombosis reflects complex interactions involving hypercoagulability, inflammation, endothelial dysfunction, treatment exposure, and metastatic dissemination [1, 24]. Previous studies have suggested that coagulation-related and inflammatory pathways may differ across tumor types and clinical contexts [25–27]. In gastric cancer, however, most mechanistic evidence remains indirect, and the currently available observational data are not sufficient to define a stable and clinically actionable thrombosis prediction framework. This may partly explain why variables that appear significant in individual studies fail to demonstrate between-study robustness when quantitatively synthesized.

The narrative synthesis of other factors yielded similarly cautious conclusions. Age, tumor stage, history of surgery, and the Khorana Risk Score did not show consistent associations across studies. Although the Khorana Risk Score is widely used for VTE risk stratification in patients with solid tumors [28], its predictive utility in gastric cancer remains uncertain. The current evidence therefore does not support routine extrapolation of general oncology risk models to this population without further validation. More broadly, the absence of stable associations in this review should not be interpreted as proof that these factors are clinically irrelevant in all gastric cancer settings. Rather, it highlights the limitations of the current evidence base and the need for better standardized study designs.

From a clinical perspective, the present findings suggest that thrombotic risk in gastric cancer across treatment settings after diagnosis should not be underestimated. However, the available evidence does not establish robust and reproducible predictors that could directly support thromboprophylaxis strategies or a gastric cancer–specific risk assessment model for clinical implementation. At present, clinical decision-making should continue to rely on comprehensive judgment that incorporates overall disease burden, treatment intensity, bleeding risk, and the broader clinical context, rather than dependence on any single reported factor. The main clinical contribution of this review is therefore to emphasize that thrombotic risk extends beyond perioperative settings and that future risk assessment in gastric cancer requires more rigorous supporting evidence.

This study has several strengths. First, it extends beyond prior perioperative-focused reviews by systematically integrating evidence on thrombotic events across treatment settings after diagnosis. Second, when data permitted, quantitative pooling was performed using multivariable-adjusted effect estimates, and robustness was explored through leave-one-out sensitivity analyses, fixed-effect comparisons, and subgroup analyses. Third, the review explicitly distinguished between studies reporting VTE alone and those also including IVT, allowing a more transparent interpretation of pooled incidence estimates across different outcome definitions.

Several limitations should also be acknowledged. Most included studies were observational, and the quantitative analyses for individual associated factors were based on a limited number of studies, resulting in reduced statistical power. Because fewer than ten studies were available for most pooled analyses, formal assessment of publication bias and small-study effects was not feasible, and these possibilities cannot be excluded. Outcome definitions were heterogeneous across studies, particularly regarding whether symptomatic events alone or both symptomatic and incidental events were included, as well as whether IVT was incorporated into the thrombotic outcome definition. The included populations also differed with respect to disease stage, treatment setting, and multimodal treatment exposure, which may have contributed to clinical heterogeneity and limited generalizability. In addition, because follow-up windows were not uniform across studies, the pooled cumulative incidence should not be interpreted as representing a standardized risk over a single fixed follow-up duration. A formal subgroup analysis by follow-up interval was not considered methodologically robust because the included studies differed not only in follow-up duration, but also in time origin and the way the observation window was defined. Finally, the high exclusion rate during study selection reflects both the broad retrieval strategy used to maximize sensitivity and the relatively specific research question of this review, which may have affected the comprehensiveness of the synthesized evidence.

Overall, relative to prior perioperative-focused reviews, this study summarizes the burden of thrombotic events in gastric cancer across treatment settings after diagnosis and synthesizes the currently available evidence regarding associated factors. Although robust and reproducible independent predictors have not yet been established, the present findings help clarify the current scope, uncertainties, and limitations of the literature. They should be understood as an evidence synthesis of reported burden and associated factors, rather than as direct support for immediate thromboprophylaxis strategies or clinical implementation.

## Conclusion

This systematic review and meta-analysis showed that the pooled incidence of thrombotic events in patients with gastric cancer across treatment settings after diagnosis was 9.0%, indicating that thrombotic risk extends beyond studies limited to the perioperative setting and should not be viewed as confined to surgical risk alone.

Although multiple associated factors were quantitatively synthesized, the current evidence does not establish any robust and reproducible independent predictors. These findings clarify both the burden of thrombotic events and the limitations of the available evidence, underscoring the need for continued clinical vigilance across treatment settings after diagnosis. However, the current evidence remains insufficient to directly support thromboprophylaxis strategies or to establish a gastric cancer–specific risk assessment model for clinical implementation.

Future large-scale prospective studies using standardized outcome definitions, screening strategies, and follow-up frameworks are needed to improve the stability and reproducibility of risk estimation and to support the future development and validation of more reliable risk assessment approaches in this population.

## Supplementary Information

Below is the link to the electronic supplementary material.

Supplementary Material 1

## Data Availability

All data analyzed in this study are derived from published articles and their supplementary materials. The datasets used and/or analyzed during the current study are available from the corresponding author upon reasonable request.

## Declarations

### Registration and protocol

This systematic review and meta-analysis was conducted in accordance with the PRISMA 2020 guidelines and was prospectively registered in the PROSPERO database (registration number: CRD420261303039).
