# Supplementary material for: Incidence and associated factors of venous thromboembolism in patients with gastric cancer across treatment settings after diagnosis: a systematic review and meta-analysis
Source: Thromb J. 2026 Apr 21;24:54. doi: 10.1186/s12959-026-00864-7 (PMC13235215; doi:10.1186/s12959-026-00864-7)

**Supplementary Table 1.**PRISMA 2020 Checklist for the Present Systematic Review and Meta-analysis

| **Section** | **Item No.** | **Checklist Item** | **Reported on Page(s)** |
| --- | --- | --- | --- |
| Title | 1 | Identify the report as a systematic review and/or meta-analysis | Title page |
| Abstract | 2 | Provide a structured summary including background, objectives, data sources, eligibility criteria, participants, interventions, methods, results, limitations, conclusions, and funding | Abstract |
| Introduction | 3 | Describe the rationale for the review in the context of existing knowledge | Introduction |
|  | 4 | Provide an explicit statement of the objectives or questions addressed with reference to participants, interventions, comparisons, outcomes, and study design (PICOS) | Introduction |
| Methods | 5 | Specify the inclusion and exclusion criteria for the review and how studies were grouped for syntheses | Methods (Eligibility criteria) |
|  | 6 | Specify all information sources (e.g., databases, registers) and the date when each was last searched | Methods (Literature search) |
|  | 7 | Present the full search strategies for all databases, including any filters and limits used | Supplementary Table 2 |
|  | 8 | Describe the process for selecting studies (screening, eligibility, inclusion) | Methods (Study selection) |
|  | 9 | Describe the process for data collection and methods of obtaining or confirming data from investigators | Methods (Data extraction) |
|  | 10a | List and define all outcomes for which data were sought | Methods (Outcomes) |
|  | 10b | List and define all other variables for which data were sought (e.g., participant characteristics, risk factors) | Methods (Risk factors) |
|  | 11 | Describe the methods used for assessing risk of bias in included studies | Methods (Quality assessment) |
|  | 12 | Specify the effect measures used for each outcome (e.g., incidence, HR, OR) | Methods (Statistical analysis) |
|  | 13a | Describe the processes used to decide which studies were eligible for each synthesis | Methods (Data synthesis) |
|  | 13b | Describe any methods required to prepare the data for presentation or synthesis | Methods (Data synthesis) |
|  | 13c | Describe the methods used to synthesize results and provide a rationale for the choice of model | Methods (Statistical analysis) |
|  | 13d | Describe any methods used to assess heterogeneity | Methods (Statistical analysis) |
|  | 13e | Describe any sensitivity analyses conducted | Methods (Statistical analysis; Sensitivity analysis) |
|  | 14 | Describe any methods used to assess reporting bias | Methods (Statistical analysis) |
|  | 15 | Describe any methods used to assess certainty of evidence | Not performed |
| Results | 16a | Describe the results of the search and study selection process, ideally using a flow diagram | Results; Figure 1 |
|  | 16b | Cite studies that appeared to meet inclusion criteria but were excluded and explain why | Figure 1 |
|  | 17 | Present characteristics of included studies | Table 1 |
|  | 18 | Present assessments of risk of bias for each included study | Table 2; Table 3 |
|  | 19 | Present results of individual studies for all outcomes | Results section |
|  | 20a | Present results of all statistical syntheses conducted | Figures 2–8 |
|  | 20b | Present results of investigations of heterogeneity | Results section |
|  | 20c | Present results of sensitivity analyses | Supplementary Figures S1–S13 |
|  | 21 | Present assessments of reporting bias | Not assessable (<10 studies per analysis) |
|  | 22 | Present assessments of certainty of evidence | Not performed |
| Discussion | 23a | Provide a general interpretation of the results in the context of other evidence | Discussion |
|  | 23b | Discuss limitations of the evidence included in the review | Discussion (Limitations) |
|  | 23c | Discuss implications of the results for practice, policy, and future research | Discussion |
| Other Information | 24a | Provide registration information for the review | Methods (Registration: PROSPERO CRD420261303039) |
|  | 24b | Indicate where the review protocol can be accessed | PROSPERO |
|  | 24c | Describe any amendments to the protocol | Not applicable |
|  | 25 | Describe sources of support (financial or non-financial) | Funding statement |
|  | 26 | Declare competing interests | Conflict of interest statement |
|  | 27 | Describe availability of data, code, and other materials | Data availability statement |

**Supplementary Table 2.** Full database-specific search strategies and dates of final search

| **Database** | **Date of final search** | **Full search strategy** |
| --- | --- | --- |
| **PubMed** | January 31, 2026 | ("Stomach Neoplasms"[Mesh] OR gastric cancer[Title/Abstract] OR stomach cancer[Title/Abstract] OR gastric neoplasm*[Title/Abstract] OR stomach neoplasm*[Title/Abstract] OR gastric carcinoma*[Title/Abstract] OR stomach carcinoma*[Title/Abstract] OR gastric adenocarcinoma*[Title/Abstract]) AND ("Venous Thromboembolism"[Mesh] OR "Venous Thrombosis"[Mesh] OR "Pulmonary Embolism"[Mesh] OR venous thromboembolism[Title/Abstract] OR VTE[Title/Abstract] OR deep vein thrombosis[Title/Abstract] OR DVT[Title/Abstract] OR pulmonary embolism[Title/Abstract] OR PE[Title/Abstract]) |
| **Embase** | January 31, 2026 | ('stomach cancer'/exp OR 'gastric cancer':ti,ab OR 'stomach cancer':ti,ab OR 'gastric neoplasm*':ti,ab OR 'stomach neoplasm*':ti,ab OR 'gastric carcinoma*':ti,ab OR 'stomach carcinoma*':ti,ab OR 'gastric adenocarcinoma*':ti,ab) AND ('venous thromboembolism'/exp OR 'venous thrombosis'/exp OR 'pulmonary embolism'/exp OR 'venous thromboembolism':ti,ab OR VTE:ti,ab OR 'deep vein thrombosis':ti,ab OR DVT:ti,ab OR 'pulmonary embolism':ti,ab OR PE:ti,ab) |
| **Web of Science** | January 31, 2026 | TS=( ("gastric cancer" OR "stomach cancer" OR "gastric neoplasm*" OR "stomach neoplasm*" OR "gastric carcinoma*" OR "stomach carcinoma*" OR "gastric adenocarcinoma*") AND ("venous thromboembolism" OR VTE OR "deep vein thrombosis" OR DVT OR "pulmonary embolism" OR PE) ) |
| **Cochrane Library** | January 31, 2026 | ("gastric cancer" OR "stomach cancer" OR "gastric neoplasm*" OR "stomach neoplasm*" OR "gastric carcinoma*" OR "stomach carcinoma*" OR "gastric adenocarcinoma*")  AND  ("venous thromboembolism" OR VTE OR "deep vein thrombosis" OR DVT OR "pulmonary embolism" OR PE)  in Title Abstract Keyword |

### Supplementary Figure S1. Leave-one-out sensitivity analysis for the pooled incidence of VTE in patients with gastric cancer


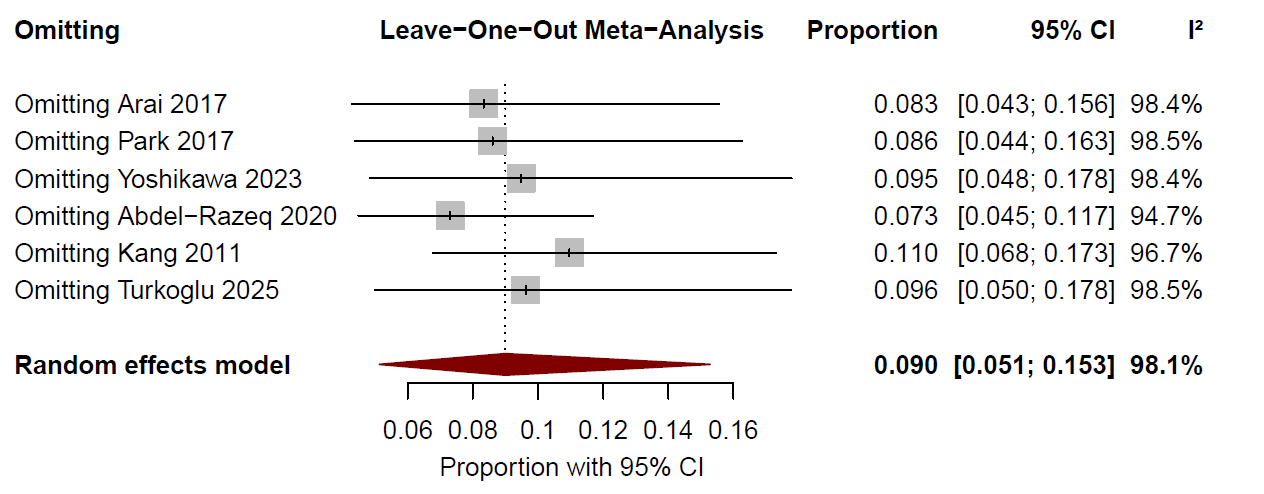


### ****Supplementary Figure S2: Fixed-effect model analysis for the pooled incidence of VTE in patients with gastric cancer****

###
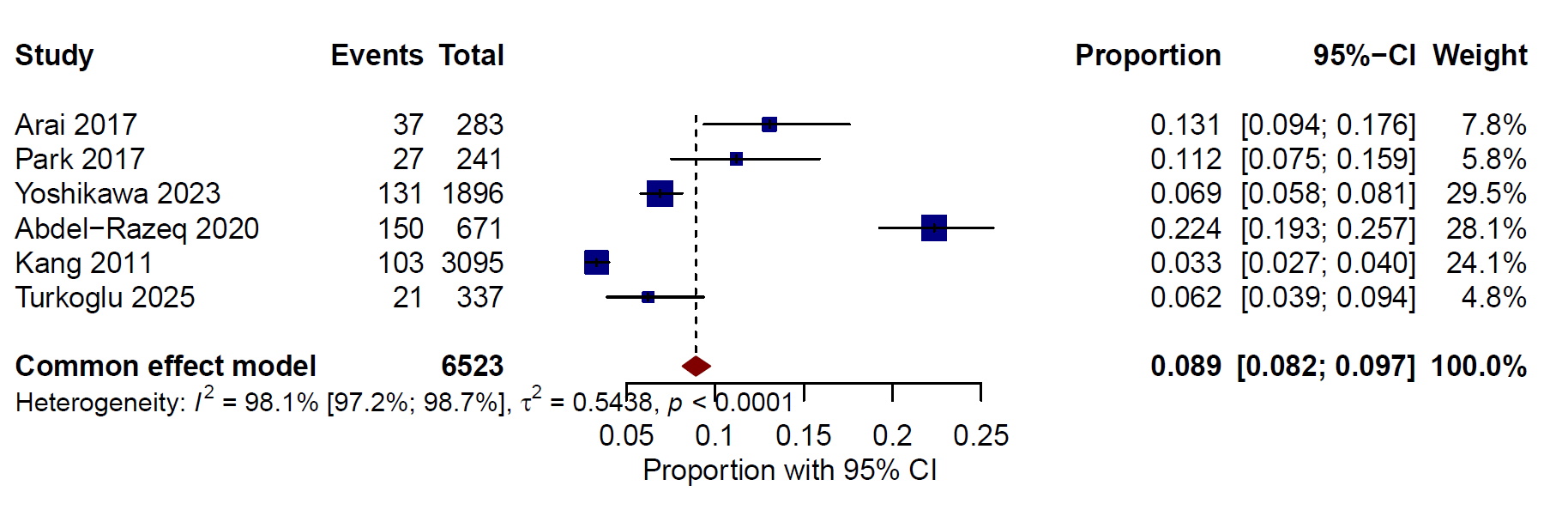


### ****Supplementary Figure S3: Leave-one-out sensitivity analysis for the association between female sex and VTE risk****


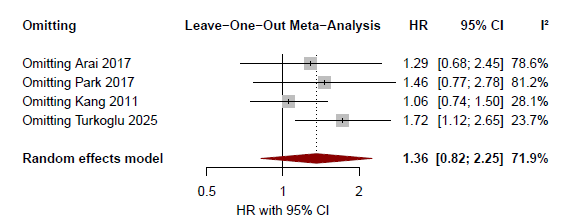


### ****Supplementary Figure S4:Fixed-effect model analysis for the association between female sex and VTE risk****


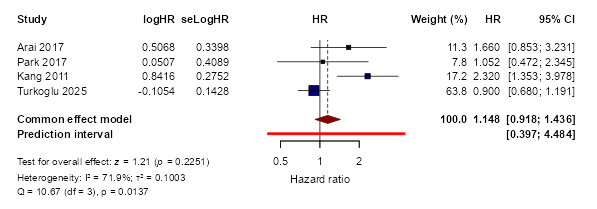


### ****Supplementary Figure S5: Leave-one-out sensitivity analysis for the association between BMI ≥25 kg/m² and VTE risk****


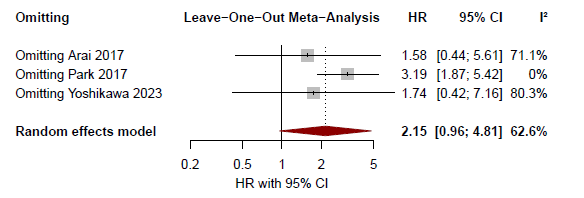


### ****Supplementary Figure S6: Fixed-effect model analysis for the association between BMI ≥25 kg/m² and VTE risk****


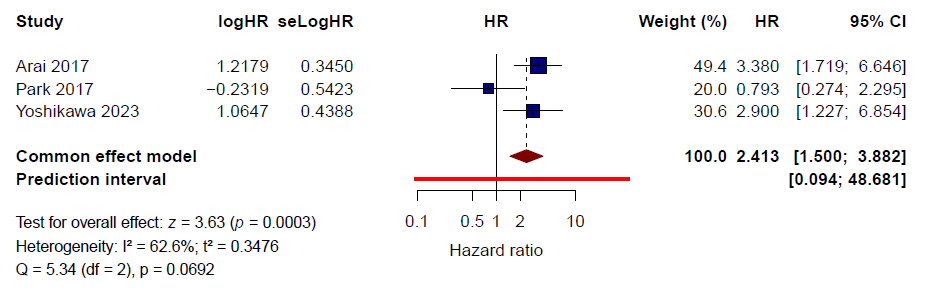


### ****Supplementary Figure S7: Leave-one-out sensitivity analysis for the association between peritoneal metastasis and VTE risk****


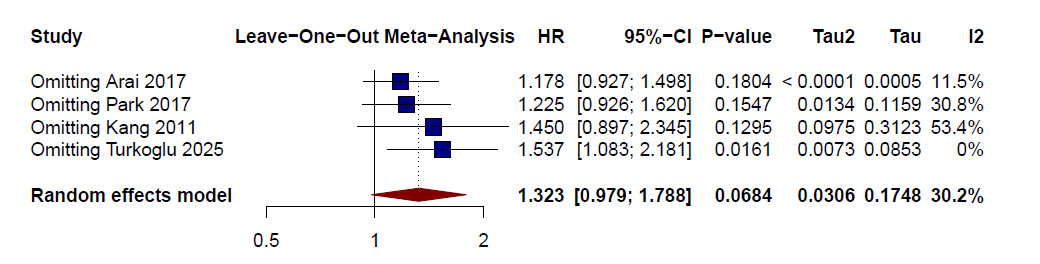


### ****Supplementary Figure S8: Fixed-effect model analysis for the association between peritoneal metastasis and VTE risk****


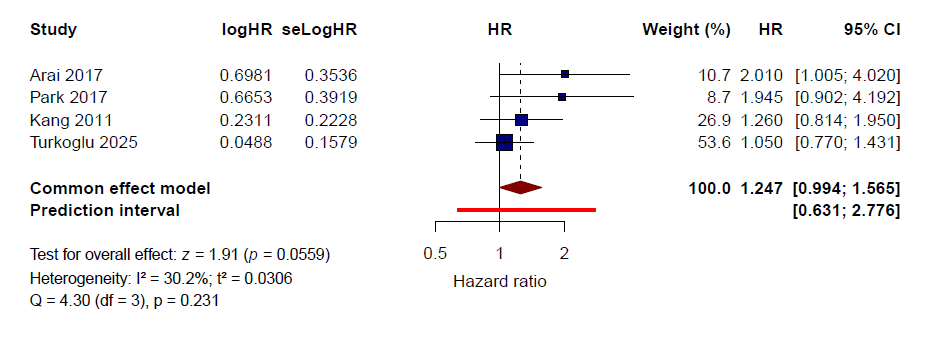


### ****Supplementary Figure S9: Leave-one-out sensitivity analysis for the association between lung metastasis and VTE risk****


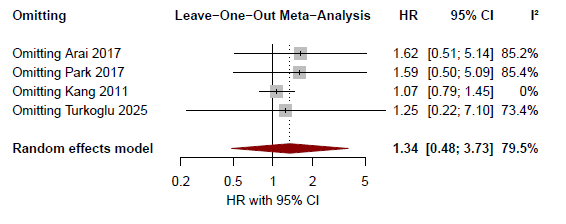


### ****Supplementary Figure S10: Fixed-effect model analysis for the association between lung metastasis and VTE risk****


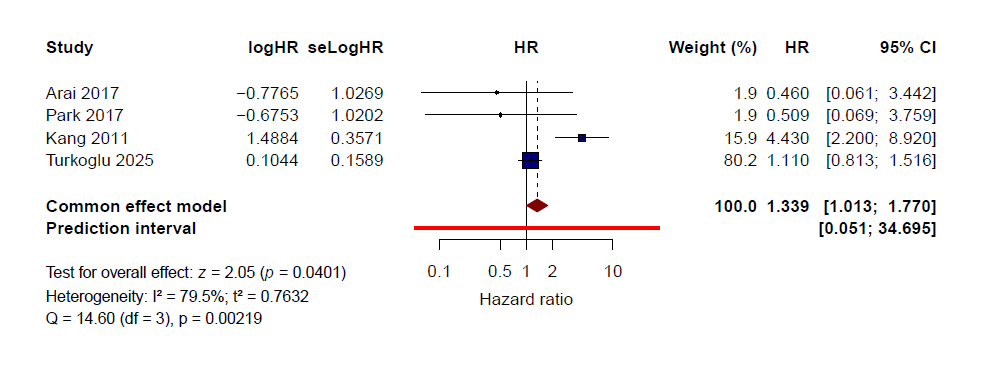


### ****Supplementary Figure S11: Leave-one-out sensitivity analysis for the association between bone metastasis and VTE risk****


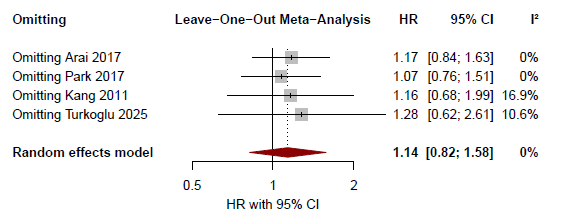


### ****Supplementary Figure S12: Fixed-effect model analysis for the association between bone metastasis and VTE risk****


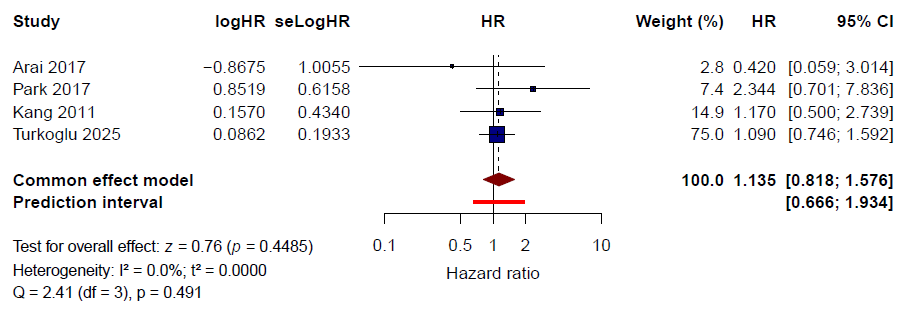

Supplement: Supplementary file 1 — Supplementary Material 1 [file 12959_2026_864_MOESM1_ESM.docx]
